# Early markers of cardiovascular disease are associated with occupational exposure to polycyclic aromatic hydrocarbons

**DOI:** 10.1038/s41598-017-09956-x

**Published:** 2017-08-25

**Authors:** Ayman Alhamdow, Christian Lindh, Maria Albin, Per Gustavsson, Håkan Tinnerberg, Karin Broberg

**Affiliations:** 1 Institute of Environmental Medicine, Karolinska Institutet, Stockholm, Sweden; 2 Division of Occupational and Environmental Medicine, Department of Laboratory Medicine, Lund University, Lund, Sweden

## Abstract

Occupational exposure to soot, rich in polycyclic aromatic hydrocarbons (PAH), has been associated with increased risk of cardiovascular disease (CVD). However, our knowledge about PAH exposure and early markers of CVD remains limited. In this cross-sectional study of 151 chimney sweeps and 152 controls, we investigated occupational exposure to PAH and early markers of CVD. Blood pressure (BP) (chimney sweeps only), urinary PAH metabolites and serum biomarkers were measured (C-reactive protein, homocysteine, gamma-glutamyltransferase, cholesterol, HDL, LDL, and triglycerides). Chimney sweeps had up to 7 times higher concentrations of PAH metabolites in urine than controls (*P* < 0.001): median concentrations (adjusted for specific gravity) for 1-hydroxypyrene, 2-hydroxyphenanthrene, 3-hydroxybenzo[a]pyrene, and 3-hydroxybenzo[a]anthracene were 0.56 µg/L, 0.78 µg/L, 4.75 ng/L, and 6.28 ng/L, respectively. Compared with controls, chimney sweeps had increased homocysteine, cholesterol, and HDL (*β* = 3.4 µmol*/*L, 0.43 mmol/L, and 0.13 mmol/L, respectively, *P* ≤ 0.003, adjusted for age, BMI, and smoking). In chimney sweeps, PAH metabolites correlated positively with the percentage of soot sweeping (*P* < 0.001). 2-hydroxyphenanthrene, 3-hydroxybenzo[a]pyrene, and 3-hydroxybenzo[a]anthracene were positively associated with diastolic BP (*P* < 0.044, adjusted for age, BMI, and smoking). PAH exposure among chimney sweeps resulted in elevated levels of markers for CVD risk. These findings stress the need to reduce occupational exposure to PAH.

## Introduction

Polycyclic aromatic hydrocarbons (PAH) are composed of two or more fused benzene rings. These omnipresent pollutants form naturally or by incomplete combustion of organic matter^1^. In addition to the well-documented carcinogenic effects of PAH^2^, exposure to PAH in the general environment and at work may contribute to development of cardiovascular disease (CVD), including coronary heart disease, peripheral arterial disease, stroke, and myocardial infarction^3–7^. For example, PAH metabolites in urine, i.e. 2-OH-PH, correlated with increased CVD events in the U.S. general population (median = 0.061 and 0.064 µg/L for the 2001-2002 and 2003-2004 surveys, respectively)^5^. Moreover, a previous study of asphalt pavers reported an increased risk for fatal ischemic heart disease in relation to occupational exposure to benzo(a)pyrene (*n* = 12,367 male asphalt pavers from 7 countries)^3^. The underlying mechanisms are not understood, but oxidative stress and systemic inflammation have been suggested to play a role in PAH-induced CVD^8, 9^. In this context, investigating early markers of CVD in relationship to PAH exposure will add important knowledge to understanding the mechanisms behind PAH-related CVD.

Humans can be exposed to PAH from many sources, such as ambient air, water, food, medication, and tobacco smoking, as well as from work^10^. Occupational exposure has been of interest because workers in several professions, such as chimney sweeping, asphalt paving, and production of aluminum and coke, could receive high PAH exposures by inhalation, dermal contact, or even contaminated food at their places of work^10^. A previous study estimated that between 1990 and 1993, about one million workers in the European Union, including 18,000 from Sweden, were exposed to PAH at concentrations higher than background levels^11^.

Chimney sweeps are primarily exposed to PAH from soot^10, 12, 13^, particularly during soot sweeping, the removal of soot from chimneys and boilers in private homes and industrial buildings that predominantly use wood, petroleum oil, and coal as fuel. Chimney sweeps can also be exposed to other hazardous substances from work such as arsenic, lead, nickel, asbestos, carbon particles, sulfur dioxide, carbon monoxide, detergents, and degreasing materials^10, 14, 15^.

Epidemiological studies have shown that, in addition to different types of cancer^16–19^, chimney sweeps have increased incidence of myocardial infarction^20^, and excess mortality from CVD^21^. Nevertheless, some factors have likely altered the chimney sweeps’ exposure to PAH over time, including changes in the type of fuel used for heat production, along with increasing use of protective equipment by chimney sweeps^22^. Therefore, characterization of the exposure to PAH and potential risk of developing CVD, by linking individual data on exposure to PAH to individual data on early markers of CVD, will provide important insight into the effects of PAH on risk of CVD among chimney sweeps working today.

Our study aimed to: 1) evaluate occupational exposure to PAH among chimney sweeps by measuring PAH metabolites in urine and identifying the work tasks that might be associated with increased exposure, and 2) estimate the risk of CVD by measuring blood pressure and early serum biomarkers for risk of CVD.

## Results

### General characteristics of participants

The general characteristics of the chimney sweeps and controls are presented in Tables 1 and 2. Both groups had comparable ages (median age 43 years), exposure to PAH from hobbies, exposure to passive smoking, levels of physical activity, education, and dietary habits, i.e., intake of vegetables, fruits, and fish. Also, the study groups did not differ in personal history of CVD, asthma, or other pulmonary diseases, kidney disease, cancer, diabetes, or family history of CVD and cancer. Chimney sweeps had significantly lower BMI and higher numbers of party smokers, snus users, as well as small-city residents, compared with controls (*P* < 0.05). Sampling season differed between groups as more controls (24%) were recruited during the summer than chimney sweeps (11%).

**Table 1:** Characteristics of study participants (all males) including serum markers related to cardiovascular disease.

	Chimney sweeps *n* = 151				Controls *n* = 152				*P* ^h^
	*n*	Median	Min	Max	*n*	Median	Min	Max	
BMI^a^ (kg/m^2^)	151	26.3	19.1	36.6	152	27.1	20.4	44.9	0.019
Age (years)	151	43	19	66	152	43	20	63	0.803
Systolic BP^b^ (mm Hg)	151	130	105	179	—	—	—	—	—^i^
Diastolic BP^b^ (mm Hg)	151	80	57	120	—	—	—	—	—^i^
CRP^c^ (mg/L)	146	0.70	0.42	46	148	1.05	0.42	19	0.040
GGT^d^ (µkat/L)	146	0.49	0.19	3.24	148	0.48	0.11	2.14	0.609
Homocysteine (µmol/L)	146	16	5.40	76	148	13	4.0	63	<0.001
Cholesterol (mmol/L)	146	5.47	3.20	8.72	148	4.97	1.96	9.70	0.005
HDL^e^ (mmol/L)	146	1.32	0.70	3.04	148	1.15	0.44	2.60	<0.001
LDL^f^ (mmol/L)	146	3.53	1.51	6.47	148	3.31	1.17	7.75	0.054
TG^g^ (mmol/L)	146	1.60	0.53	8.40	148	1.70	0.41	7.40	0.670

^a^Body mass index, ^b^blood pressure, ^c^C-reactive protein, ^d^gamma-glutamyl transferase, ^e^high-density lipoprotein, ^f^low-density lipoprotein, ^g^triglycerides. ^h^ *P*-value for Mann-Whitney U test. ^i^No comparison has been performed because blood pressure was only available for chimney sweeps.

**Table 2:** Characteristics of study participants including lifestyle factors and personal and family disease histories.

Variables	Chimney sweeps *n* = 151	Controls *n* = 152	*P* ^f^
	*n* ^e^ (%)	*n* ^e^ (%)	
Smoking (smoker/party-/ex-/non-smoker)	27/25/24/74 (18/17/16/49)	25/4/40/83 (16/3/26/55)	<0.001
Snus (yes/no)	52/98 (35/65)	29/123 (19/81)	0.003
Passive smoking (yes/no)	28/121 (19/81)	24/128 (16/84)	0.543
Sampling season (spring/summer/autumn/winter)	47/16/71/16 (31/11/47/11)	51/36/52/13 (33/24/34/9)	0.011
Residency (big city/small city/big town/small town)	20/32/60/37 (13/22/40/25)	37/33/59/23 (24/22/39/15)	0.039
Education (high school or lower/university or higher)	126/24 (84/16)	120/31 (79/21)	0.371
Physical activity (high/low)	73/77 (49/51)	62/90 (41/59)	0.218
Exposure from hobby (yes/no)	9/141 (6/94)	6/146 (4/96)	0.441
Vegetables^a^ (≥5 times a week/< 5 times a week)	96/54 (64/36)	88/63 (58/42)	0.345
Fruits^b^ (≥5 times a week/<5 times a week)	75/75 (50/50)	88/63 (58/42)	0.202
Fish^c^ (≥ once a week/< once a week)	73/77 (49/51)	70/82 (46/54)	0.484
Myocardial infarction (yes/no)	4/146 (3/97)	0 (0)	0.060
Angina pectoris (yes/no)	7/143 (5/95)	9/142 (6/94)	0.798
Hypertension (yes/no)	27/123 (18/82)	22/129 (15/85)	0.439
Stroke (yes/no)	1/149 (1/99)	2/150 (1/99)	1.000
Thrombosis in arm/leg (yes/no)	6/144 (4/96)	3/149 (2/98)	0.334
Other heart disease (yes/no)	3/147 (2/98)	3/149 (2/98)	1.000
All CVD-related morbidities^d^ (yes/no)	39/111 (26/74)	35/116 (23/77)	0.594
Kidney/ urinary disease (yes/no)	7/143 (5/95)	6/146 (4/96)	0.785
Diabetes (yes/no)	1/149 (1/99)	6/146 (4/96)	0.121
Family history of CVD (yes/no)	68/78 (47/53)	54/94 (36/64)	0.097

^a^Intake of all kinds of vegetables, legumes and root vegetables (fresh, frozen, canned, stewed, juice, soup, etc.). ^b^Intake of all kinds of fruits and berries (fresh, frozen, canned, juice, jam, etc.). ^c^Intake of all kinds of fish. ^d^Having current or previous cardiovascular disease(s), i.e. myocardial infarction, angina pectoris, hypertension, stroke, thrombosis in arm/leg and other heart diseases. ^e^There were up to 5 missing cases for some of the variables. ^f^ *P*-value for Fisher’s Exact test.

CVD-related morbidities (myocardial infarction, angina pectoris, hypertension, stroke, thrombosis) in chimney sweeps did not differ from controls (*P* = 0.594). However, it is worth mentioning that among chimney sweeps there were 4 cases of myocardial infarction but none among controls.

### The chimney sweeping profession has changed over time

**Figure 1:**
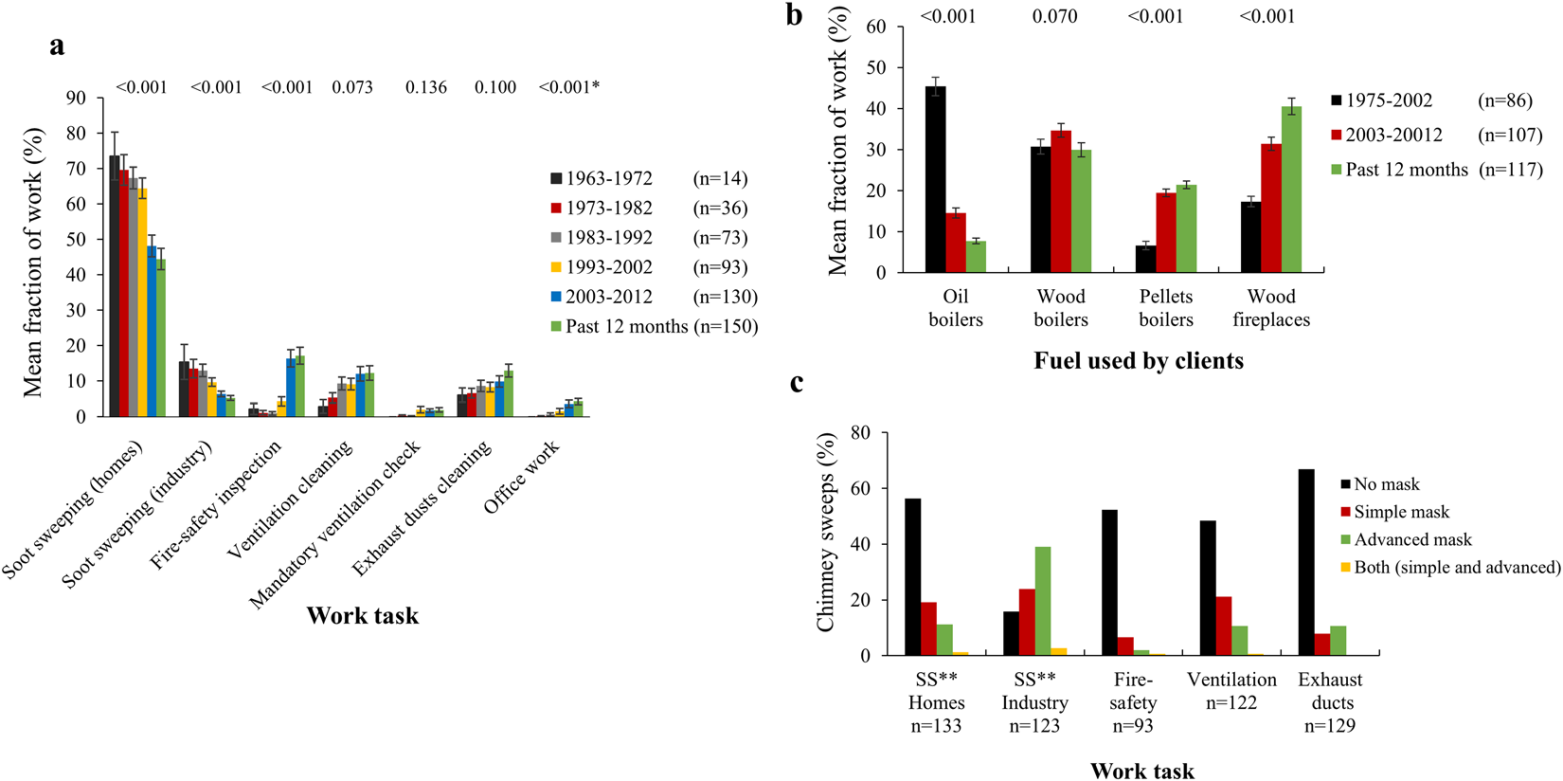
Trends for work tasks, use of fuel by clients, and use of masks in chimney sweeps. (**a**) Fraction of work (%) for different work tasks from 1963 to 2013. (**b**) Fraction of work (%) with different types of fuels used by clients from 1975 to 2013. (**c**) Percentage of chimney sweeps that used masks (simple and/or advanced) during work for five different work tasks. *Wilcoxson signed rank test for work tasks before/after the year 2002. **Soot sweeping.

Soot sweeping in private homes was the prevailing task for chimney sweeps with 44% of the sweeping time spent during the past 12 months (Fig. 1a). Comparing before and after the year 2002, soot sweeping in both private homes and industrial buildings significantly decreased, whereas, inspection of fire-safety systems and office work increased (*P* < 0.001). The use of fuel before and after 2002 changed, as the use of petroleum oil significantly decreased and the use of pellets and wood (for fireplaces) increased (*P* < 0.001, Fig. 1b). Chimney sweeps used personal protective devices to a varying extent; 10–20% of the chimney sweeps did not use gloves, and 50–85% did not use masks during soot sweeping (Supplementary Table S1 and Fig. 1c).

### Occupational exposure to soot is associated with higher levels of PAH metabolites in urine

**Figure 2:**
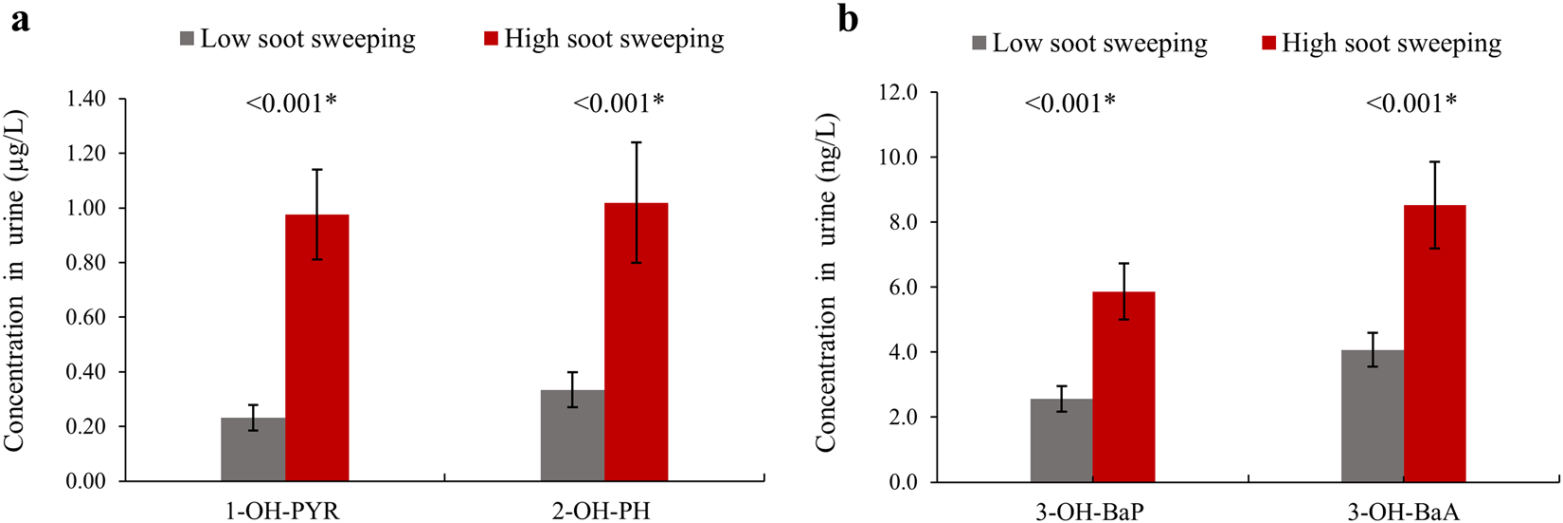
Differences in PAH metabolites between chimney sweeps (exposed group) that spent less than 50% (low soot sweeping) and those who spent 50% or more (high soot sweeping) of their working time doing total soot sweeping (private homes and industry in the past 12 months). (**a**) PAH metabolites 1-hydroxypyrene (1-OH-PYR) and 2-hydroxyphenanthrene (2-OH-PH). (**b**) PAH metabolites 3-hydroxybenzo[a]pyrene (3-OH-BaP) and 3-hydroxybenzo[a]anthracene (3-OH-BaA). Standard errors for PAH metabolites are represented by whiskers on top of each bar. *P-value for general linear model adjusted for age, BMI and smoking status.

PAH metabolite concentrations in urine for chimney sweeps and controls are shown in Table 3. Chimney sweeps had significantly higher concentrations of 1-OH-PYR, 2-OH-PH, 3-OH-BaP, and 3-OH-BaA compared with controls (*P* < 0.001 for all metabolites, adjusted for age, smoking, and BMI). The median values for chimney sweeps were 7-fold higher for 1-OH-PYR, 4-fold higher for 2-OH-PH, and 3-fold higher for 3-OH-BaP and 3-OH-BaA, compared with controls (specific gravity-adjusted concentrations). Comparable figures were seen for creatinine-adjusted concentrations (Supplementary Table S2). The sampling season or the day of sampling did not affect the results; therefore, it was excluded from adjustment in model 3. After excluding current and party smokers, the levels of the four metabolites were highly correlated in the chimney sweep group (*P* < 0.001, *r*_*S*_ >0.68 for all), but not in the control group (Supplementary Table S3). In addition, soot sweeping in private homes during the past 12 months positively correlated with all metabolites (*P* < 0.001, *r*_*S*_ ≥0.52 for all); however, the correlations were weak for soot sweeping in industrial buildings (Supplementary Table S4a). Chimney sweeps who spent half of their working time or more doing soot sweeping in private homes and industrial buildings during the past 12 months had up to 4 times higher PAH metabolite levels compared with sweeps that worked less with soot sweeping (*P* < 0.001, adjusted for age, BMI, and smoking, Fig. 2). Also in general linear models, PAH metabolites were associated with soot sweeping (continuous variable) during the last 12 months (1-OH-PYR *β* = 0.16 95%CI 0.11, 0.20; 2-OH-PH *β* = 0.16 95%CI 0.10, 0.23; 3-OH-BaP *β* = 0.71 95%CI 0.45, 0.98; and 3-OH-BaA *β* = 1.06 95%CI 0.66, 1.4; *P* < 0.001, adjusted for age, BMI, and smoking). Non-soot sweeping tasks showed negative associations with PAH metabolites. We further evaluated the potential association between PAH metabolites and the use of gloves and masks during soot sweeping in the past 12 months but no significant associations were seen (Supplementary Table S4b).

**Table 3:** Concentrations of urinary metabolites (specific gravity adjusted) of polycyclic aromatic hydrocarbons among exposed and unexposed groups.

All participants	Chimney sweeps				Controls				
	*n* ^e^	Median	Min	Max	*n* ^e^	Median	Min	Max	*P* ^f^
1-OH-PYR^a^	148	0.56	0.03	7.49	151	0.07	0.00	1.25	<0.001
2-OH-PH^b^	148	0.78	0.09	14.56	151	0.18	0.05	4.01	<0.001
3-OH-BaP^c^	132	4.75	0.00	42.86	130	1.38	0.00	16.51	<0.001
3-OH-BaA^d^	142	6.28	0.46	71.70	144	1.99	0.04	20.88	<0.001
**Only non-smokers**									
1-OH-PYR	95	0.50	0.03	7.17	123	0.07	0.005	0.53	<0.001
2-OH-PH	95	0.68	0.09	14.56	123	0.16	0.05	4.01	<0.001
3-OH-BaP	90	4.18	0.00	31.89	105	1.18	0.00	16.51	<0.001
3-OH-BaA	92	5.53	0.46	71.70	117	1.80	0.04	8.12	<0.001

^a^1-hydroxypyrene (µg/L), ^b^2-hydroxyphenanthrene (µg/L), ^c^3-hydroxybenzo[a]pyrene (ng/L). ^d^3-hydroxybenzo[a]anthracene (ng/L). ^e^The number of samples with valid concentrations for 3-OH-BaP and 3-OH-BaA was lower, compared with other two metabolites, due to their low concentrations in urine. ^f^General linear model adjusted for age, BMI and smoking status.

### Occupational exposure to soot is associated with markers of risk of CVD

In general linear models, chimney sweeps had higher levels of homocysteine (*P* ≤ 0.001), HDL (*P* ≤ 0.004) and cholesterol (*P* ≤ 0.010) compared with controls (Table 4). The effect estimates did not differ much between different models. LDL, TG, and GGT levels showed higher, but non-significant, effect estimates for chimney sweeps in all models (*P* ≥ 0.061). Chimney sweeps had lower non-significant CRP levels compared with controls.

**Table 4:** Differences between chimney sweeps and control group in serum markers of cardiovascular disease, explored with a general-linear-model. Effect estimates are presented as *β*-values [95%CI = 95% confidence interval].

Variable	Model 1^f^		Model 2^g^		Model 3^h^	
	*P*	*β*1 (CI 95%)	*P*	*β*1 (CI 95%)	*P*	*β*1 (CI 95%)
CRP^a^	0.709	−0.16 (−0.98, 0.67)	0.938	0.04 (−0.98, 1.06)	0.747	−0.15 (−1.04, 0.75)
GGT^b^	0.193	0.06 (−0.03, 0.16)	0.497	0.04 (−0.07, 0.14)	0.201	0.07 (−0.04, 0.17)
Homocysteine	<0.001	3.37 (1.67, 5.07)	<0.001	2.86 (1.36, 4.37)	0.001	2.96 (1.18, 4.74)
HDL^c^	0.004	0.13 (0.04, 0.22)	0.011	0.13 (0.03, 0.23)	0.001	0.16 (0.06, 0.26)
Cholesterol	0.003	0.43 (0.15, 0.72)	0.015	0.39 (0.08, 0.71)	0.005	0.44 (0.13, 0.75)
LDL^d^	0.061	0.25 (−0.01, 0.51)	0.225	0.18 (−0.11, 0.47)	0.070	0.26 (−0.02, 0.55)
TG^e^	0.190	0.19 (−0.10, 0.48)	0.074	0.26 (−0.03, 0.54)	0.446	0.12 (−0.19, 0.43)

^a^C-reactive protein, ^b^gamma-glutamyl transferase, ^c^high-density lipoprotein, ^d^low-density lipoprotein, ^e^triglycerides. ^f^(serum marker) = intercept + β1 × exposure group (2 categories) + β2 × age (continuous) + β3 × BMI (continuous) + β4 × smoking status (4 categories) + e (residual error). ^g^(serum marker) = intercept + β1 × exposure group (2 categories) + β2 × age (continuous) + β3 × BMI (continuous) + e (residual error). Current and party smokers were excluded from this analysis. ^h^(serum marker) = intercept + β1 × exposure group (2 categories) + β2 × age (continuous) + β3 × BMI (continuous) + β4 × smoking status (4 categories) + β5 × use of snus (2 categories) + β6 × physical activity (4 categories) + β7 × passive smoking (2 categories) + β8 × current residence (4 categories) + β9 × education (2 categories) + β10 × family history of cardiovascular disease (2 categories) + β11 × exposure to polycyclic aromatic hydrocarbons from hobby (2 categories) + e (residual error).

In the chimney sweeps, 2-OH-PH, 3-OH-BaP, and 3-OH-BaA were positively associated with diastolic blood pressure in model 1 and 2 (*P* ≤ 0.044). However, the association remained significant only for 2-OH-PH in the fully adjusted model (Table 5). Further, associations between PAH metabolites in urine and the serum markers homocysteine, HDL, and cholesterol were weak (Table 5). Homocysteine was positively associated with all metabolites, but it was only significant for 3-OH-BaA among non-smokers (Table 5, model 2). For HDL, overall negative associations were found with the PAH metabolites, but it was only significant for 3-OH-BaA in model 1. No associations were found for cholesterol.

**Table 5:** Associations between PAH metabolites in urine and blood pressure in the exposed group (chimney sweeps, *n* = 151), explored by general-linear-model [95%CI = 95% confidence interval].

**Systolic BP** ^**a**^	Model 1^f^		Model 2^g^		Model 3^h^	
	*P*	*β*1 (CI 95%)	*P*	*β*1 (CI 95%)	*P*	*β*1 (CI 95%)
1-OH-PYR^b^	0.702	0.37 (−1.55, 2.29)	0.872	0.22 (−2.46, 2.90)	0.512	0.72 (−1.45, 2.89)
2-OH-PH^c^	0.247	0.87 (−0.61, 2.36)	0.223	1.02 (−0.63, 2.67)	0.071	1.58 (−0.14, 3.29)
3-OH-BaP^d^	0.359	0.18 (−0.21, 0.57)	0.521	0.20 (−0.41, 0.81)	0.369	0.20 (−0.24, 0.63)
3-OH-BaA^e^	0.766	0.04 (−0.21, 0.29)	0.406	0.13 (−0.18, 0.44)	0.549	0.09 (−0.20, 0.38)
**Diastolic BP** ^**a**^						
1-OH-PYR	0.067	1.16 (−0.08, 2.40)	0.219	1.08 (−0.65, 2.82)	0.151	1.00 (−0.37, 2.37)
2-OH-PH	0.001	1.66 (0.72, 2.60)	<0.001	1.88 (0.86, 2.89)	0.002	1.72 (0.66, 2.78)
3-OH-BaP	0.013	0.31 (0.07, 0.55)	0.016	0.47 (0.09, 0.85)	0.107	0.22 (−0.05, 0.49)
3-OH-BaA	0.044	0.16 (0.01, 0.32)	0.015	0.24 (0.05, 0.44)	0.089	0.16 (−0.03, 0.34)
**Homocysteine**						
1-OH-PYR	0.668	0.27 (−0.98, 1.53)	0.138	0.70 (−0.23, 1.64)	0.897	−0.09 (−1.49, 1.30)
2-OH-PH	0.389	0.38 (−0.49, 1.25)	0.121	0.46 (−0.12, 1.04)	0.520	0.32 (−0.66, 1.29)
3-OH-BaP	0.262	0.11 (−0.09, 0.32)	0.363	0.10 (−0.12, 0.31)	0.170	0.15 (−0.06, 0.36)
3-OH-BaA	0.264	0.09 (−0.07, 0.24)	0.030	0.12 (0.01, 0.23)	0.508	0.06 (−0.11, 0.23)
**Cholesterol**						
1-OH-PYR	0.789	0.02 (−0.15, 0.20)	0.880	0.02 (−0.20, 0.23)	0.851	0.02 (−0.19, 0.22)
2-OH-PH	0.619	0.03 (−0.09, 0.15)	0.637	0.03 (−0.10, 0.17)	0.723	0.03 (−0.12, 0.17)
3-OH-BaP	0.932	0.00 (−0.04, 0.04)	0.631	0.01 (−0.04, 0.06)	0.892	0.00 (−0.04, 0.05)
3-OH-BaA	0.612	0.01 (−0.02, 0.03)	0.675	0.01 (−0.02, 0.03)	0.613	0.01 (−0.02, 0.03)
**HDL**						
1-OH-PYR	0.123	−0.05 (−0.10, 0.01)	0.501	−0.03 (−0.10, 0.05)	0.381	−0.03 (−0.10, 0.04)
2-OH-PH	0.073	−0.04 (−0.08, 0.00)	0.221	−0.03 (−0.07, 0.02)	0.298	−0.02 (−0.07, 0.02)
3-OH-BaP	0.067	−0.01 (−0.02, 0.00)	0.296	−0.01 (−0.03, 0.01)	0.223	−0.01 (−0.02, 0.01)
3-OH-BaA	0.042	−0.01 (−0.01, 0.00)	0.224	−0.01 (−0.01, 0.00)	0.185	−0.01 (−0.01, 0.00)

^a^Blood pressure. ^b^1-hydroxypyrene (µg/L). ^c^2-hydroxyphenanthrene (µg/L). ^d^3-hydroxybenzo[a]pyrene (ng/L). ^e^3-hydroxybenzo[a]anthracene (ng/L). ^f^(outcome variable) = intercept + β1 × PAH metabolite (continuous) + β2 × age (continuous) + β3 × BMI (continuous) + β4 × smoking status (4 categories) + e (residual error). ^g^(outcome variable) = intercept + β1 × PAH metabolite (continuous) + β2 × age (continuous) + β3 × BMI (continuous) + e (residual error). Current and party smokers were excluded from the analysis. ^h^(outcome variable) = intercept + β1 × PAH metabolite (continuous) + β2 × age (continuous) + β3 × BMI (continuous) + β4 × smoking status (4 categories) + β5 × use of snus (2 categories) + β6 × physical activity (4 categories) + β7 × passive smoking (2 categories) + β8 × current residence (4 categories) + β9 × education (2 categories) + β10 × family history of cardiovascular disease (2 categories) + β11 × exposure to polycyclic aromatic hydrocarbons from hobby (2 categories) + e (residual error).

Working years of soot sweeping was not more strongly associated with blood pressure or serum markers (homocysteine, HDL and cholesterol) than age (Supplementary Table S5).

## Discussion

Our study shows that chimney sweeps who are currently working are exposed to PAH from work: urinary PAH metabolites were up to 7 times higher in chimney sweeps than in controls, and working with soot sweeping correlated with concentrations of PAH metabolites. These findings demonstrate that chimney sweeps are still exposed to hazardous PAH, despite the fact that the use of oil has declined since 1975 and soot sweeping in homes and industrial buildings declined during the same time. Alarmingly, the use of protective equipment during work only increased to a small extent over the same time period. The trends for exposure and use of protective equipment are in line with our previous work on the chimney sweep profession^22^. Furthermore, chimney sweeps showed an increased risk for CVD represented in higher serum concentrations of homocysteine and cholesterol compared with controls. The reason for the elevated risk of CVD may be sweeps’ exposure to PAH, since one PAH metabolite correlated weakly with homocysteine and three PAH metabolites in urine correlated with increased diastolic blood pressure and among chimney sweeps.

Our study had noteworthy advantages and shortcomings. To our knowledge, this is the first study relating individual data on PAH exposure to individual data on early markers of CVD in chimney sweeps. We recruited currently working chimney sweeps and controls, and collected extensive information on their work and lifestyle, including potential confounders. We measured the urinary metabolites of PAH using a sensitive, reliable technique, LC-MS/MS. However, the measurement of urinary 3-OH-BaP and 3-OH-BaA was hampered due to low concentrations in urine. The 3-OH-BaP concentrations are usually 1000 times lower in urine than 1-OH-PYR^23^. Studies on rats showed that the primary route of excretion for benzo[a]pyrene was via feces and only 0.1–0.3% of benzo[a]pyrene is excreted in urine as 3-OH-BaP upon intravenous administration^24, 25^. Yet, the PAH metabolites showed strong correlations in the chimney sweep group, particularly between 1-OH-PYR and 2-OH-PH, as well as between 1-OH-PYR and 3-OH-BaA, supporting the quality of our analysis and indicating common sources of exposure, i.e. exposure to soot. We did not have information for the day or week of sampling about the percentage of time spent with each work task, use of protective equipment, and diet, which could have confounded metabolite concentrations.

Cigarette smoking is a major source of PAH exposure for the general population^10, 26, 27^ and therefore, we conducted the statistical analyses after excluding smokers. The medians for PAH metabolite concentrations were slightly lower after excluding current and party smokers. Still, chimney sweeps had up to 7 times higher concentrations of PAH metabolites compared with controls, confirming that their work results in high exposure to PAH. When stratifying for current place of residence, living in big cities did not seem to affect PAH exposure. In contrast, chimney sweeps living in smaller cities had higher concentrations of PAH metabolites and controls showed no difference in PAH metabolites between cities. This could be explained by the fact that chimney sweeps living in smaller cities worked more with soot sweeping, the major source of exposure to PAH. We did not have data on the intake of grilled, broiled, smoked, and fried food for the day or week of sampling. Those food items might have introduced additional exposure to PAH that we did not control for. However, there was no difference in the intake of vegetables, fruits and fish between groups and sampling season did not affect the concentrations of PAH metabolites. Further studies should take airborne measurements of PAH^28^ into account and dietary intake of PAH.

Another issue is that PAH exposure might have been associated with other occupational or environmental toxicants (e.g., particles, combustion gases, and degreasing chemicals) that can potentially lead to CVD^29^ and we cannot exclude that some of the differences in serum markers between chimney sweeps are related to concurrent exposures. In addition, chimney sweeps’ recall bias might have affected the answers to the questionnaire, as we retrospectively asked chimney sweeps about the percentage of time spent with each work task and their use of protective equipment throughout different time periods. Also, the recruitment for controls may not have been optimal, since it was done in two different years, 2011 and 2015.

Exposure to PAH is considerable among chimney sweeps in other countries and in other professions, suggesting that many workers are at risk for CVD-related changes: the 1-OH-PYR concentrations in our study (median: 0.56, 0.03–7.49 µg/L) were lower than those reported by Letzel *et al*. (median: 0.7, <0.1–12.8 µg/L, chimney sweeps from Germany and Poland), Göen *et al*. (median: 1.03, 0.15–3.97 µg/L, from Germany) and Pavanello *et al*. (mean: 1.59, 0.11–6.65 µg/L, from Italy)^10, 12, 30–32^. Workers at aluminum plants, coke ovens, and foundries, as well as workers laying asphalt pavement, experience considerable exposure to PAH. The median concentrations for 1-OH-PYR in urine ranged from 0.3 µg/L in foundry workers to 7.0 µg/L in coke oven workers^33^.

Compared with the majority of other professions where studies have produced data on 3-OH-BaP levels in urine, the chimney sweeps in our study showed relatively high 3-OH-BaP (3.35 among all and 2.9 ng/g creatinine among non-smoking chimney sweeps): median concentrations of 3-OH-BaP in urine were 1.1 ng/g creatinine in fireproof material production, 0.5 ng/g creatinine in coke oven plants, 1.3 ng/g creatinine in graphite electrode plants, 1.2 ng/g creatinine in converter infeed, below LOQ in steel plants, and 14 ng/g creatinine in fireproof stone plants^34–36^. This is of concern as benzo[a]pyrene is an established carcinogen in humans (IARC 2010) and our findings suggest that current chimney sweeps may be at risk for cancer as well as CVD.

The strong positive correlations between PAH metabolites and soot sweeping in the past 12 months were unexpected as PAH metabolites generally have short half-lives (3.9–35 h) and therefore only reflect recent exposure to the parent compounds^37–41^. A likely explanation would be that chimney sweeps reported the sampling’s week figures for the percentage of time for each task as a projection for the past 12-month period.

Among chimney sweeps, we found some dose-response relationships with markers of CVD. PAH metabolites (2-OH-PH, 3-OH-BaP, 3-OH-BaA) consistently associated with diastolic blood pressure when adjusted for age, BMI, and smoking (or excluding smokers). 2-OH-PH showed the strongest association and the highest effect estimates. For every 1 µg/L increase in urinary 2-OH-PH, diastolic blood pressure increased by 1.7 mm Hg. That dramatic increase implies higher risk for developing future CVD among chimney sweeps, as observational studies with more than 1 million total participants have shown that every 10 mm Hg increment in diastolic blood pressure was associated with a 2-fold increase in mortality from CVD^42^. In line with our finding, urinary 2-OH-PH concentrations were associated with increased incidence of hypertension in a population-based study on 4765 adults^43^. Working years with soot sweeping was highly correlated with age and it was therefore difficult to evaluate the effect of working years on blood pressure. However, when comparing the effect of age and working years on blood pressure separately, we could not see a clear influence of working years of soot sweeping. There are no clear mechanisms linking PAH exposure with changes in blood pressure, but it has been proposed that PAH can affect the autonomic nervous system^44^.

Also, PAH metabolites were positively associated with homocysteine, although significance was only reached in the non-smokers for one of the metabolites, suggesting that PAH impairs one-carbon metabolism. No dose-response effect was found for cholesterol with PAH metabolites despite the fact that the chimney sweeps had elevated serum concentrations of homocysteine and cholesterol compared with controls. One possibility for this finding is that other exposures, e.g. particles or degreasing chemicals, could have caused the elevation of cholesterol. Nevertheless, higher homocysteine and cholesterol is of major concern since there is strong evidence linking these biomarkers with adverse cardiovascular events^45–52^. Increased homocysteine levels can also be driven by several factors, e.g. vitamin B deficiency, atherosclerotic events, medication, and diabetes^48^. We did not have data on vitamin B deficiency, but we evaluated the differences in homocysteine concentrations after excluding participants with diabetes and those using regular medication. Still, chimney sweeps had higher homocysteine levels in serum than controls. The observation that the chimney sweep group had higher levels of HDL compared with controls was intriguing, but when we restricted the analysis to chimney sweeps, we only found negative associations between HDL and PAH metabolites (model 1). This suggests that the higher HDL among chimney sweeps compared with controls is not linked to soot exposure. Studies in the general population have shown associations between PAH metabolites and increasing levels of GGT and CRP^53–55^ and a study on 489 coke oven workers showed a positive association between PAH metabolites and plasma CRP^56^. However, in our study the levels of other serum biomarkers i.e., GGT, CRP, LDL and TG were not different between chimney sweeps and controls.

## Conclusions

In conclusion, currently working chimney sweeps showed clear occupational exposure to PAH. Chimney sweeps had a higher risk for developing CVD, indicated by elevated homocysteine and cholesterol levels in serum. Our study shows that PAH metabolites were associated with increased diastolic blood pressure, suggesting exposure to PAH as a driving factor for developing CVD. These findings warrant further investigation of the cardiovascular profile of workers exposed to PAH. They also suggest the need for incentives to increase the proper use of protective measures during work and to develop different work routines.

## Material and Methods

### Study groups

We enrolled 151 male chimney sweeps and 152 male controls from southern Sweden. Chimney sweeps were recruited from 29 different companies. Every chimney sweep performs multiple tasks: soot sweeping, white sweeping (i.e., inspection of fire-safety systems, cleaning ventilation channels, mandatory inspection of ventilation channels, and cleaning exhaust-ducts in restaurants), and office work. Their work usually starts in the morning when chimney sweeps drive to their client’s premises, carried out the sweeping task, and then returned to their company to shower and do task-related office work. The controls were employees at ten companies (i.e., food storage and supermarkets, housing, waste management, goods transport) and four municipalities, and were not occupationally exposed to PAH.

### Recruitment

Between 2013 and 2015, we contacted, in coordination with the chimney sweeps’ trade union, managers of 39 chimney-sweeping companies to ask for participation in the study; 34 of them agreed, resulting in 87% company response rate. Five of the 34 companies were in the end not included in the study: one company had only female employees (n = 2, the only female sweepers in the study) and four companies were located far away and for logistic reasons not included in the study, leaving 29 companies. We instructed the manager at each company to invite all employees that currently were working.

Thereafter we booked a visit at the company for a general medical examination and sampling of blood and urine of chimney sweeps that had agreed to participate. We sent informed consent forms and questionnaires to each participant in advance. The visits at the companies took place after the chimney sweeps had finished their shift on Wednesdays or Thursdays. On the day of visit, a trained nurse checked that the questionnaires were completed, sampled blood (non-fasting sample) and urine, and measured blood pressure, height, and weight of the participants.

For the nonsmoking control group, we directly contacted the companies’ managers without involving the trade union. As a result, 7 companies were positive for participation out of 12, ending up with 58% company response rate for the nonsmoking controls. We instructed the manager at each company to invite all employees that currently were working. The controls were recruited during two periods: 127 nonsmoking controls were recruited in 2010–11 and 25 smokers in 2015. The smoking controls (3 companies and 4 municipalities) were introduced into the study to match the smoking status of the chimney sweeps. The controls also filled in a questionnaire, donated blood and urine, and had their blood pressure, height, and weight measured.

All participants gave informed consent before participation in the study. The study was approved by the Regional Ethics Committee at Lund University, Lund, Sweden and we confirm that all experiments were performed in accordance with relevant guidelines and regulations.

### Questionnaire

The questionnaire for chimney sweeps and controls contained questions about age, weight, height, education (5 categories, from primary school to university or higher), personal history of disease and symptoms (yes/no) (myocardial infarction, angina pectoris, hypertension, stroke, thrombosis, asthma, chronic obstructive lung disease, chronic cough with phlegm, severe cough, infectious diseases, kidney and urinary disease, diabetes, and cancer), family history of CVD and cancer (yes/no), prescribed medicines (yes/no), over-the-counter medicines (yes/no), dietary habits regarding consumption of vegetables, fruits, and fish (8 categories, from none to 3 times a day or more), physical activity (4 categories, from inactive to very active), tobacco smoking (3 categories, from nonsmoker to current smoker), passive smoking (yes/no), use of snus (yes/no), current residency (4 categories, from big city to small town), employment history, and exposure from hobby (smoke, fumes, diesel exhaust, engine exhaust, dust).

In addition, the questionnaire given to the chimney sweeps enquired about the details of chimney sweeping tasks (i.e., “soot sweeping in private homes”, “soot sweeping in industrial buildings”, “inspection of fire-safety systems”, “cleaning ventilation channels”, “mandatory inspection of ventilation channels”, “cleaning exhaust ducts in restaurants” and “office work”). The questions about the aforementioned tasks concerned 6 periods (i.e., 1963–72, 1973–82, 1983–92, 1993–2002, 2003–12, and the past 12 months). For instance, “what was the extent of soot sweeping in private homes on a scale from 0 to 100% in the past 12 months, considering that the total percentage for all tasks must be 100%”. Chimney sweeps were also asked, on a 0–100% scale, to what extent that their clients used different types of fuels (petroleum oil, wood, wood pellets) in private homes or industrial buildings during 3 different periods (1975–2002, 2003–12, and the past 12 months). Over the same 3 periods (1975–2002, 2003–12, and the past 12 months), the sweeps were asked to estimate how often they used gloves, masks, long-sleeved shirts, long pants, and protective overall suits, on a scale from 0–100% for each exposure-related task (i.e., soot sweeping in private homes, soot sweeping in industrial buildings, inspection of fire-safety systems, cleaning ventilation channels, cleaning exhaust ducts in restaurants). However, questions about the use of vacuum machines concerned only soot sweeping in private homes and industrial buildings. Furthermore, information about the frequency of changing gloves (daily, once/week, once/month, rarely) and type of mask (simple, advanced) was collected.

### Blood pressure measurement and sampling

Blood pressure was measured using a mercury sphygmomanometer in the upright (sitting) position for chimney sweeps (*n* = 151) after a rest of at least 15 min. Post-shift spot urine samples and venous blood samples were collected from chimney sweeps and controls (non-fasting samples). Buffy coat, EDTA, and heparin plasma, and serum were separated after 10 minutes and kept on dry ice until storage at −80 °C at the Division of Occupational and Environmental Medicine at Lund University, whereas urine and EDTA blood samples were kept at room temperature until storage at −20 °C. Nine participants abstained from giving blood samples (5 chimney sweeps and 4 controls) and 4 participants could not urinate during the visit (3 chimney sweeps and 1 control).

### Exposure assessment by measurement of PAH metabolites in urine

Total exposure to PAH was evaluated by analyzing 4 different PAH metabolites in urine. These metabolites have been suggested as biomarkers for PAH exposure. 1-hydroxypyrene (1-OH-PYR) is a metabolite of pyrene and has been extensively used as a proxy for total exposure to PAH. For exposure to benzo[a]pyrene, we measured the metabolite 3-hydroxybenzo[a]pyrene (3-OH-BaP)^34^; for exposure to phenanthrene and benzo[a]anthracene, we measured the metabolites 2-hydroxyphenanthrene (2-OH-PH) and 3-hydroxybenzo[a]anthracene (3-OH-BaA)^36^, respectively.

Liquid chromatography coupled to tandem mass spectrometry (LC-MS/MS; QTRAP 5500, AB Sciex, Foster City, CA, USA) was used for measurement of PAH metabolites in urine. The urine samples were prepared in 96-well plates and hydrolysed using glucuronidase. Internal standards for all PAH metabolites were added. For analysis of 1-OH-PYR and 2-OH-PH, sample aliquots of 5 µL were injected onto a C18 column^57^. For analysis of 3-OH-BaA and 3-OH-BaP, sample aliquots of 20 µL were injected onto a two-dimensional LC system with two analytical columns. Concentrations were determined by peak area ratios of the analytes versus the internal standards. All samples were prepared in duplicate and the average concentration of the duplicate samples was used. For more details, see Supplementary Methods and Supplementary Table S6.

A hand refractometer was used to measure the specific gravity (SG) of urine. PAH metabolite concentrations in urine (C) were adjusted for SG according to C_adjusted_ = C_(__measured__)_ × (1.020 − 1)/(ρ − 1), where C_(__measured__)_ was the determined concentration in a urine sample, ρ was the measured SG for the same sample, and 1.020 was the average SG of all urine samples in this study. Urinary creatinine was measured by an enzymatic colorimetric method^58^. We adjusted PAH metabolites in urine to the creatinine concentrations for comparison with other studies (Supplementary Table S2).

### Biomarkers for cardiovascular disease

Aliquots from serum samples that had not been previously thawed were analyzed for seven different early biomarkers of CVD at the Department of Clinical Chemistry, Lund University Hospital. C-reactive protein (CRP), homocysteine, and gamma-glutamyltransferase (GGT) have been reported to be elevated in multiple CVD events^48, 49, 59, 60^. CRP was quantified by using monoclonal CRP antibodies and by measuring the absorbance with immunoturbidimetry (limit of detection (LOD) <0.6 mg/L). Homocysteine was measured by an enzymatic reaction followed by quantification of the absorbance of NAD^+^ at 340 nm (LOD <3 µmol/L). GGT was determined by a method based on formation of 5-amino-2-nitrobenzoate, which was measured by kinetic photometry (LOD <0.05 µkat/L). Biomarkers of blood lipid status (low-density lipoprotein (LDL), high-density lipoprotein (HDL), cholesterol, and triglycerides (TG)) were also considered for analysis of dyslipidemia, which is a traditional risk factor for CVD^46, 47, 52, 61^. LDL was measured by selective micellar solubilization (LOD <0.1 mmol/L); HDL by an enzymatic colorimetric method (LOD <0.08 mmol/L); cholesterol by a method based on formation a quinone dye measured photometrically (LOD <0.1 mmol/L); and TG by a method based on lipase and peroxidase (LOD <0.1 mmol/L). The levels of CRP, homocysteine, and LDL in serum had previously been determined in the non-smoking controls, but here we performed new measurements of these markers using fresh (not previously thawed) aliquots of the same serum samples. The correlations between old and new measurements were high (*r*_*S*_ = 0.83; 0.88 and 0.95 for homocysteine, LDL, and CRP, respectively), which strengthens our analysis.

### Statistical analyses

Age was calculated based on the birth and recruitment dates. BMI was obtained using the formula BMI = weight in kilograms/(height in meters)^2^. For cigarette smoking, participants were categorized into 4 groups: smoker (daily smoker), party smoker (party smoker or ex-smoker who quit smoking less than a year before the day of recruitment), ex-smoker (quit smoking more than a year before the day of recruitment) and non-smoker (never smoked). For PAH metabolite concentrations below the LOD, measured concentrations were used. For CVD marker concentrations below the LOD, the values were replaced by LOD/√2. Median, minimum, and maximum values were calculated for continuous variables and frequencies for categorical ones.

The Mann-Whitney U test was used to preliminarily compare differences in continuous variables between chimney sweeps and controls, i.e., BMI, age, and serum markers of CVD. Fisher’s Exact test was used to compare differences in distribution of categorical variables between groups. Spearman’s correlations were used to assess the correlations between PAH metabolites and between PAH metabolites and work tasks, use of gloves and masks during the past 12 months. Changes over time in work tasks, use of fuel by clients, and use of protective equipment were explored by Wilcoxon signed-rank tests having the year 2002 as an arbitrary cutoff.

A general linear model was used to evaluate differences in CVD serum markers and PAH metabolites between chimney sweeps and controls, adjusting for potential covariates and confounders such as age, BMI, and smoking, which are well-known risk factors for CVD, as well as other lifestyle factors that could cause additional exposure to PAH. We performed the analyses in three different models: 1) adjusted for age (continuous), BMI (continuous), and smoking status (smoker, party smoker, ex-smoker, non-smoker); 2) adjusted for age and BMI (excluding current and party smokers); and 3) adjusted for age, BMI, smoking status, use of snus (yes/no), physical activity (4 categories; from low to regular exercise), passive smoking (yes/no), current residency (big city, small city, big town, small town), education (2 categories; high school or lower, higher than high school), family history of CVD (yes/no) and exposure to PAH from hobby (yes/no). A general linear model (adjusted for age, BMI, and smoking) was also used to evaluate whether working with soot sweeping correlated with PAH metabolites in urine. The sweeps were divided into two groups based on the percentage of time spent doing soot sweeping in private homes and industrial buildings in the past 12 months (i.e., ≥50% vs. <50% of their working time with soot sweeping).

In the chimney sweep group only, the same 3 models were adopted to assess associations between PAH metabolites and markers of CVD. We calculated the dose of exposure to soot over time (1963–2013), expressed as working years with soot sweeping, as follows: percentage of work with soot sweeping during the past 12 months divided by 100, plus percentage of work with soot sweeping in every 10-year work period divided by 10. Calculations were performed for the sum of soot sweeping in private homes and industrial buildings. For instance, one chimney sweep spent 20% of his work time doing soot sweeping in private homes and industrial buildings during the past 12 months, 40% during 2003–2012 and 80% during 1993–2002. Working years with soot sweeping for this participant would then be (0.2*1 + 0.4*10 + 0.8*10), which equals 12.2 years. We investigated the effect of working years with soot sweeping on blood pressure and serum markers using general linear model analysis as follows: first, we evaluated age versus blood pressure/serum markers (excluding working years from the models), and second, we evaluated working years versus blood pressure/serum markers (excluding age from the models).

Statistical analysis was performed using SPSS 22.0 (SPSS Inc, Chicago, IL, USA). *P*-values < 0.05 were considered statistically significant (two-tailed).

### Data availability statement

All data generated or analyzed during this study are included in this article and its supplementary information files.

## Electronic supplementary material

Supplemental material
