# Supplementary material for: Early markers of cardiovascular disease are associated with occupational exposure to polycyclic aromatic hydrocarbons
Source: Sci Rep. 2017 Aug 25;7:9426. doi: 10.1038/s41598-017-09956-x (PMC5573323; doi:10.1038/s41598-017-09956-x)
Supplement: Supplementary file 1 — Supplemental material [file 41598_2017_9956_MOESM1_ESM.pdf]

Supplemental Material

Early markers of cardiovascular disease are associated with occupational exposure to polycyclic aromatic hydrocarbons

Ayman Alhamdow[1], Christian Lindh[2], Maria Albin[1,2], Per Gustavsson[1], Håkan Tinnerberg[2], Karin Broberg*[1,2]

[1]Institute of Environmental Medicine, Karolinska Institutet, Stockholm, Sweden

[2]Division of Occupational and Environmental Medicine, Department of Laboratory Medicine, Lund University, Lund, Sweden

*Correspondence to: Karin Broberg

Postal address: Unit of Metals & Health, Institute of Environmental Medicine, Karolinska Institutet, Nobels Väg 13, Stockholm 171 77, Sweden

E-mail: karin.broberg@ki.se

Table of Contents

Supplementary Methods for measurement of polycyclic aromatic hydrocarbon (PAH) metabolites in urine

- Chemicals
- Instrumentation
- Sample preparation
- Instrumental Analysis
- Validation

Supplementary Table S1. Use of protective clothing and equipment by chimney sweeps from 1975–2013 (mean percentage), stratified by different work tasks

Supplementary Table S2. Urinary metabolites of polycyclic aromatic hydrocarbons (adjusted for creatinine) among chimney sweeps and controls

Supplementary Table S3. Correlations between polycyclic aromatic hydrocarbon metabolites in chimney sweeps and controls (current and party smokers were excluded), evaluated by Spearman's correlation

Supplementary Table S4a. Spearman's correlations for polycyclic aromatic hydrocarbon metabolites and work tasks (chimney sweeps) during the past 12 months

Supplementary Table S4b. Spearman's correlations for polycyclic aromatic hydrocarbon metabolites and use of gloves and masks during soot sweeping (homes and industrial buildings) during the past 12 months

Supplementary Table S5. Associations between A) age and B) working years with soot sweeping and blood pressure (BP) and serum markers among chimney sweeps, explored by general linear model [95 %CI=95% confidence interval]

Supplementary Table S6. SRM transitions for 1-OH-PYR, 2-OH-PH, 3-OH-BaA, and 3-OH-BaP in LC-MS/MS analysis, including declustering potentials (DP) and collision energies (CE)

Supplementary Methods for measurement of polycyclic aromatic hydrocarbon (PAH) metabolites in urine

Chemicals

1-hydroxypyrene (1-OH-PYR), 2-hydroxyphenanthrene (2-OH-PH), 3-hydroxybenzo[a]anthracene (3-OH-BaA), 3-hydroxybenzo[a]pyrene (3-OH-BaP) and the internal standards $^{2}H_{9}$-1-OH-PYR, $^{2}H_{9}$-2-OH-PH, $^{2}H_{11}$-3-OH-BaA and $^{2}H_{11}$-3-OH-BaP were obtained from Toronto Research Chemicals, Inc. (North York, Canada). Ammonium acetate and methanol were from Merck (Darmstadt, Germany). Water was from a Milli-Q Integral 5 system (Millipore, Billerica, MA, USA). β-Glucuronidase (*Escherichia coli* K12) was obtained from Roche Diagnostics (Mannheim, Germany).

Urine samples for calibration and for quality control (QC) were obtained from healthy volunteers at our laboratory and PAH-exposed subjects. The QC samples for 1-OH-PYR had concentrations of 6 and 26 nmol/L and for 2-OH-PH 20 and 95 nmol/L.

Instrumentation

The quantitative analysis used a triple quadrupole linear ion trap mass spectrometer (MS) equipped with an electrospray ion source (QTRAP 5500; AB Sciex, Foster City, CA, USA) coupled to a liquid chromatograph with four pumps (LC-MS/MS; Shimadzu Corporation, Kyoto, Japan). Air was used as nebulizer and auxiliary gas, pure nitrogen as curtain and collision gas. The MS analyses were carried out by selected reaction monitoring in the negative mode (Supplementary Table S6).

Sample preparation

For analysis of PAH metabolites, 200 µL duplicate urine samples were placed in 1 mL micro inserts in 96-well plates, and 10 µL glucuronidase and 100 µL 1 mol/L ammonium acetate buffer, pH 6.5, were added. The samples were kept at 37°C for 30 min. After incubation, 40 µl vitamin C solution (0.18 mg/mL) and an IS solution containing $^{2}H_{9}$-1-OH-PYR, $^{2}H_{9}$-2-OH-PH, $^{2}H_{11}$-3-OH-BaA, and $^{2}H_{11}$-3-OH-BaP were added. The samples were stored at -20°C until analysis. The samples were shaken and centrifuged at 3000 rpm for 10 min just prior to analysis.

Instrumental Analysis

For analysis of 1-OH-PYR and 2-OH-PH, sample aliquots of 5 µL were injected onto a C18 column (Genesis Lightn, 2.1 mm i.d. x 100 mm, Genesis, Grace Vydac, Hesperia, CA, USA) kept at 60°C. A mobile phase gradient, with a flow rate of 0.3 mL/min, consisting of water (A) and methanol (B) was kept at 5% B for 1 min after injection, raised to 95% B in 6.7 min, kept there for 2.3 min; and for the next injection, the column was conditioned at 5% B for 3 min. A diverter valve was used to introduce the column effluent into the mass spectrometer between 5.8 and 6.9 min. The ion source temperature was 700°C; for other parameters see Supplementary Table S6.

For analysis of 3-OH-BaA and 3-OH-BaP, a two-dimensional separation was carried out, using two analytical columns: column I: Genesis (C8, 4.6 × 20 mm, 4 µm) and column II: Genesis Lightn (C18, 4.6 × 100 mm, 4 µm,), and four LC pumps. The columns and LC pumps were connected through a diverter valve. The two mobile phases used consisted of water (A) and methanol (B). An aliquot of 20 µL of the sample was injected on column I and the separation was carried out by gradient elution, beginning with 55% mobile phase B for 1.55 min and a gradient to 70% B for 1.5 min. After 2.85 min, the diverter valve switched over and the effluent was diverted onto column II for 1.3 min. The second set of pumps continued the gradient from 70% B to 95% B for 2 minutes on column II. A valve on the MS diverted the column II effluent to the MS between 4.5–6.3 min. Column I was reconditioned with 95% mobile phase B for 1 min, followed by equilibration with 55% mobile phase A for 2.5 min and column II was reconditioned with 95% mobile phase B for 1 min in the end of the analytical run and then equilibrated with 70% mobile phase B during the beginning of the next analytical run. The columns were maintained at 60°C and the flow rate was 0.6 mL/min. The ion source temperature was 700°C, for other parameters see Supplementary Table S6.

Data were acquired and processed using the supplied software (Analyst 1.6.1, Multiquant 2.1, AB Sciex). Concentrations were determined by peak area ratios of the analytes versus the ISs. Within each analytical batch, 80 urine samples, a calibration curve, 2 QC samples for 1-OH-PYR and 2-OH-PH and 2 chemical blanks were included. All samples were prepared in duplicate and analyzed by single injections. The average concentration of the duplicate samples was used.

A hand refractometer was used for specific gravity (SG) determinations of urine, the concentrations were adjusted for urinary SG according to C_{SG} = C(observed) × (1.020 − 1)/(ρ-1), where C(observed) was the determined concentration in a urine sample, ρ the measured SG, and 1.020, the average SG of all urine samples in this study.

Validation

The limit of detection (LOD) was estimated from the blank samples and it was 50 pg/mL for 1-OH-PYR, and 2-OH-PH, and 2 pg/mL for 3-OH-BaP and 3-OH-BaA. Analytical reproducibility, expressed as coefficient of variation (CV) in *n* = 400 duplicate urine samples, was 14% for 1-OH-PYR at 0.5 ng/mL, and 9% for 2-OH-PH at 0.8 ng/mL.

The between-run precision determined for 1-OH-PYR was determined from 2 QC samples and was 8% at level 1 and 6 ng/mL. The QC samples were analysed 32 times during 11 months. The between-run precision for 2-OH-PH was determined from 2 QC samples and was 9% at level 4 and 20 ng/mL. These QC samples were analysed 25 times during 8 months. The analyses of 1-OH-PYR were part of a round robin inter-laboratory programme (University of Erlangen-Nuremberg, Germany) with results within the tolerance limits.

Supplementary Table S1. Use of protective clothing and equipment by chimney sweeps from 1975–2013 (mean percentage), stratified by different work tasks

		n	SS[a] Homes	*n*	SS[b] Industry	*n*	Fire-safety[c]	*n*	Venti-lation[d]	*n*	Exhaust ducts[e]
Gloves	1975–2002	88	79.1	84	87.0	45	43.0	71	43.5	74	37.9
	2003–2012	112	80.9	107	89.1	68	49.5	101	47.0	104	46.5
	Past 12 months	129	82.4	124	90.1	76	51.7	111	46.5	120	52.1
	P-value[f]	82	0.105	79	0.003	45	0.011	69	0.249	70	0.003
Masks	1975–2002	92	11.1	85	45.6	70	2.0	78	9.2	82	1.5
	2003–2012	119	13.6	109	54.4	85	6.5	107	16.2	114	6.3
	Past 12 months	131	14.9	119	52.5	91	6.3	119	15.1	131	10.6
	P-value	90	0.021	80	0.072	70	0.043	77	0.065	81	0.068
Long sleeves	1975–2002	90	82.2	85	90.8	47	74.9	76	75.6	78	78.6
	2003–2012	115	76.8	110	86.5	70	70.6	108	69.2	113	73.3
	Past 12 months	128	74.3	122	85.9	76	70.0	115	68.0	126	71.2
	P-value	84	0.008	81	0.438	47	0.500	72	0.046	75	0.225
Long pants	1975–2002	90	99.4	85	99.8	48	92.9	77	99.7	78	99.9
	2003–2012	115	96.2	110	96.9	71	90.0	108	95.4	112	94.9
	Past 12 months	128	97.3	122	96.9	75	92.2	116	96.2	127	95.8
	P-value	84	0.180	81	0.317	48	0.276	73	0.102	75	0.180
Protective overall suits	1975–2002	80	1.9	81	16.4	62	1.9	69	3.5	75	5.0
	2003–2012	105	0.1	109	19.5	82	1.2	100	2.1	103	4.6
	Past 12 months	118	0.0	120	15.7	90	1.1	111	1.0	112	3.0
	P-value	78	0.180	79	0.183	61	1.000	67	0.043	72	0.051
Vacuum machines	1975–2002	92	63.6	85	62.8		-		-		-
	2003–2012	111	75.2	106	68.7		-		-		-
	Past 12 months	125	80.5	120	69.1		-		-		-
	P-value	84	<0.001	78	0.013		-		-		-

[a] Soot sweeping in private homes, [b] soot sweeping in industrial buildings, [c] inspection of fire-safety systems, boilers, and furnace, [d] cleaning ventilation channels in houses, buildings and industry, [e] cleaning exhaust ducts in restaurants, [f] *P*-value of Wilcoxon signed-rank test before/after the year 2002.

Supplementary Table S2. Urinary metabolites of polycyclic aromatic hydrocarbons (adjusted for creatinine) among chimney sweeps and controls

	Chimney sweeps				Controls				
**All participants**	*n*	Median	Min	Max	*n*	Median	Min	Max	P^a
1-OH-PYR (μg/g creatinine)	148	0.39	0.02	8.77	151	0.06	0.00	0.73	<0.001
2-OH-PH (μg/g creatinine)	148	0.57	0.08	7.10	151	0.14	0.04	3.09	<0.001
3-OH-BaP (ng/g creatinine)	132	3.35	0.00	50.15	130	1.03	0.00	17.28	<0.001
3-OH-BaA (ng/g creatinine)	142	4.78	0.39	43.35	144	1.66	0.04	21.02	<0.001
**Only non-smokers**									
1-OH-PYR (μg/g creatinine)	95	0.36	0.02	5.84	123	0.05	0.00	0.44	<0.001
2-OH-PH (μg/g creatinine)	95	0.50	0.08	7.10	123	0.12	0.05	3.09	<0.001
3-OH-BaP (ng/g creatinine)	90	2.88	0.00	16.23	105	0.88	0.00	17.28	<0.001
3-OH-BaA (ng/g creatinine)	92	4.16	0.39	34.96	117	1.51	0.04	6.84	<0.001

[a] General linear model adjusted for age, BMI, and smoking status.

Supplementary Table S3. Correlations between polycyclic aromatic hydrocarbon metabolites in chimney sweeps and controls (current and party smokers were excluded), evaluated by Spearman's correlation

**Chimney sweeps**		1-OH-PYR[d]	2-OH-PH	3-OH-BaP
2-OH-PH[a]	r_S	0.87		
	P-value	<0.001		
	n	95		
3-OH-BaP[b]	r_S	0.76	0.68	
	P-value	<0.001	<0.001	
	n	90	90	
3-OH-BaA[c]	r_S	0.85	0.82	0.75
	P-value	<0.001	<0.001	<0.001
	n	92	92	90
**Controls**				
2-OH-PH	r_S	0.50		
	P-value	<0.001		
	n	123		
3-OH-BaP	r_S	-0.11	-0.07	
	P-value	0.263	0.496	
	n	105	105	
3-OH-BaA	r_S	0.09	0.22	0.37
	P-value	0.330	0.015	<0.001
	n	117	117	105

[a] 2-hydroxyphenanthrene (µg/L), [b] 3-hydroxybenzo[a]pyrene (ng/L), [c] 3-hydroxybenzo[a]anthracene (ng/L), [d] 1-hydroxypyrene (µg/L).

Supplementary Table S4a. Spearman's correlations for polycyclic aromatic hydrocarbon metabolites and work tasks (chimney sweeps) during the past 12 months

Task	Correlation	1-OH-PYR[b]	2-OH-PH[c]	3-OH-BaP[d]	3-OH-BaA[e]
Soot sweeping in private homes	r_S	0.68	0.59	0.58	0.52
	P-value[a]	<0.001	<0.001	<0.001	<0.001
	n	147	147	131	141
Soot sweeping in industrial buildings	r_S	0.28	0.20	0.14	0.18
	P-value	0.001	0.014	0.120	0.031
	n	147	147	131	141
Inspection of fire-safety systems	r_S	-0.20	-0.17	-0.10	-0.19
	P-value	0.015	0.034	0.244	0.023
	n	147	147	131	141
Cleaning ventilation channels	r_S	-0.20	-0.20	-0.15	-0.19
	P-value	0.013	0.014	0.087	0.025
	n	147	147	131	141
Mandatory ventilation inspection	r_S	-0.23	-0.18	-0.19	-0.10
	P-value	0.004	0.030	0.028	0.228
	n	147	147	131	141
Cleaning exhaust ducts in restaurants	r_S	-0.07	-0.04	-0.13	-0.09
	P-value	0.379	0.604	0.146	0.297
	n	147	147	131	141
Office work	r_S	-0.27	-0.24	-0.27	-0.17
	P-value	0.001	0.004	0.002	0.038
	n	147	147	131	141

[a] Two-tailed, [b] 1-hydroxypyrene, [c] 2-hydroxyphenanthrene, [d] 3-hydroxybenzo[a]pyrene, [e] 3-hydroxybenzo[a]anthracene.

Supplementary Table S4b. Spearman's correlations for polycyclic aromatic hydrocarbon metabolites and use of gloves and masks during soot sweeping (homes and industrial buildings) during the past 12 months

Task	Correlation	1-OH-PYR[b]	2-OH-PH[c]	3-OH-BaP[d]	3-OH-BaA[e]
Gloves (private homes)	r_S	0.07	0.11	-0.05	0.11
	P-value[a]	0.463	0.208	0.619	0.213
	n	127	127	116	124
Gloves (industrial buildings)	r_S	0.11	0.12	0.01	0.14
	P-value	0.225	0.199	0.948	0.125
	n	122	122	111	119
Mask (private homes)	r_S	-0.09	-0.10	0.05	-0.16
	P-value	0.304	0.256	0.619	0.078
	n	129	129	117	125
Mask (industrial buildings)	r_S	-0.01	0.07	-0.02	0.01
	P-value	0.954	0.480	0.804	0.957
	n	117	117	105	113

[a] Two-tailed, [b] 1-hydroxypyrene, [c] 2-hydroxyphenanthrene, [d] 3-hydroxybenzo[a]pyrene, [e] 3-hydroxybenzo[a]anthracene.

Supplementary Table S5. Associations between A) age and B) working years with soot sweeping and blood pressure (BP) and serum markers among chimney sweeps, explored by general linear model [95 %CI=95% confidence interval]

**A (age)**	Model 1[a]		Model 2[b]		Model 3[c]	
	P	*β1* (CI 95%)	*P*	*β1* (CI 95%)	*P*	*β1* (CI 95%)
Systolic BP	0.134	0.15 (-0.05, 0.36)	0.046	0.24 (0.01, 0.48)	0.246	0.14 (-0.10, 0.37)
Diastolic BP	0.021	0.15 (0.02, 0.29)	0.037	0.16 (0.01, 0.32)	0.135	0.11 (-0.03, -0.26)
Homocysteine	0.120	0.09 (-0.03, 0.21)	0.011	0.11 (0.03, 0.19)	0.227	0.08 (-0.05, 0.21)
Cholesterol	0.005	0.02 (0.01, 0.04)	0.062	0.02 (0.00, 0.04)	0.009	0.03 (0.01, 0.05)
HDL	0.318	0.00 (0.00, 0.01)	0.114	0.01 (0.00, 0.01)	0.178	0.00 (0.00, 0.01)

**B (working years with soot sweeping)**	Model 1[d]		Model 2[e]		Model 3[f]	
	P	*β1* (CI 95%)	*P*	*β1* (CI 95%)	*P*	*β1* (CI 95%)
Systolic BP	0.505	0.06 (-0.13, 0.26)	0.057	0.21 (-0.01, 0.43)	0.614	0.06 (-0.18, 0.30)
Diastolic BP	0.036	0.13 (0.01, 0.26)	0.009	0.19 (0.05, 0.33)	0.164	0.11 (-0.04, 0.26)
Homocysteine	0.198	0.07 (-0.04, 0.18)	0.052	0.08 (0.00, 0.16)	0.306	0.07 (-0.06, 0.20)
Cholesterol	0.062	0.02 (0.00, 0.03)	0.100	0.02 (0.00, 0.03)	0.105	0.02 (0.00, 0.04)
HDL	0.509	0.00 (0.00, 0.01)	0.240	0.00 (0.00, 0.01)	0.109	0.01 (0.00, 0.01)

[a] BP = intercept + β1 x age (continuous) + β2 x BMI (continuous) + β3 x smoking status (4 categories) + e (residual error)
[b] BP = intercept + β1 x age (continuous) + β2 x BMI (continuous) + e (residual error). Current and party smokers excluded.

[c] BP = intercept + β1 x age (continuous) + β2 x BMI (continuous) + β3 x smoking status (4 categories) + β4 x use of snus (2 categories) + β5 x physical activity (4 categories) + β6 x passive smoking (2 categories) + β7 x current residence (4 categories) + β8 x education (2 categories) + β9 x family history of cardiovascular disease (2 categories) + β10 x exposure from hobby (2 categories) + e (residual error)

[d] BP = intercept + β1 x working years with soot sweeping (continuous) + β2 x BMI (continuous) + β3 x smoking status (4 categories) + e (residual error)

[e] BP = intercept + β1 x working years with soot sweeping (continuous) + β2 x BMI (continuous) + e (residual error). Current and party smokers excluded.

[f] BP = intercept + β1 x working years with soot sweeping (continuous) + β2 x BMI (continuous) + β3 x smoking status (4 categories) + β4 x use of snus (2 categories) + β5 x physical activity (4 categories) + β6 x passive smoking (2 categories) + β7 x current residence (4 categories) + β8 x education (2 categories) + β9 x family history of cardiovascular disease (2 categories) + β10 x exposure from hobby (2 categories) + e (residual error)

Supplementary Table S6. SRM transitions for 1-OH-PYR, 2-OH-PH, 3-OH-BaA, and 3-OH-BaP in LC-MS/MS analysis, including declustering potentials (DP) and collision energies (CE)

**Analyte**	**Transitions [m/z]**		**DP** (V)	**CE** (eV)
1-OH-PYR[a]	217 - 189	*Quantifier ion*	-140	-47
$^{2}H_{9}$-1-OH-PYR	226 - 198	*IS*	-140	-47
2-OH-PH[b]	193 - 165	*Quantifier ion*	-140	-46
$^{2}H_{9}$-2-OH-PH	203 - 174	*IS*	-140	-46
3-OH-BaA[c]	243 - 215	*Quantifier ion*	-140	-47
$^{2}H_{11}$-3-OH-BaA	254 - 226	*IS*	-140	-47
3-OH-BaP[d]	267 - 239	*Quantifier ion*	-140	-54
$^{2}H_{11}$-3-OH-BaP	278 - 250	*IS*	-140	-54

[a] 1-hydroxypyrene, [b] 2-hydroxyphenanthrene, [c] 3-hydroxybenzo[a]anthracene, [d] 3-hydroxybenzo[a]pyrene.
